# Nutrient Content of Different Wheat and Maize Varieties and Their Impact on Metabolizable Energy Content and Nitrogen Utilization by Broilers

**DOI:** 10.3390/ani10050907

**Published:** 2020-05-23

**Authors:** Olga Lasek, Jan Barteczko, Justyna Barć, Piotr Micek

**Affiliations:** Department of Animal Nutrition and Biotechnology, and Fisheries, Agricultural University of Krakow, Al. Mickiewicza 24/28, 30-059 Krakow, Poland; olga.lasek@urk.edu.pl (O.L.); kzzip@urk.edu.pl (J.B.); justyna.barc@urk.edu.pl (J.B.)

**Keywords:** broiler chickens, wheat, maize, cereal grain, carbohydrate structure, energy value

## Abstract

**Simple Summary:**

The current standard for the determination of the energy value of feed is to use regression equations; however, these equations are imprecise and may affect the correct estimation of energy for several reasons. First of all, these equations include the values of some raw components, such as crude protein, ether extracts, and N-free extracts, but not different forms of fiber, which in high concentration reduces the energy value of poultry feed. In addition, in the tables, there are average values for different feeds, which do not take into account differences between varieties of the same species, which, at least in case of grains may be much larger than differences between two different species. Another important aspect is that the concentration of various components, including antinutritional factors, affects their mutual use and thus the energy value of feed. This work was aimed at improving the precision of estimating the energy value of poultry feed by incorporating detergent and dietary fiber as well as additional nutrients such as starch and total sugars separately. In addition, the authors suggest considering the values characteristic of selected grain in the energy calculations rather than the mean values for the species, because it may improve the precision of the result.

**Abstract:**

The study aimed to determine the effect of nutrients of wheat (nine cultivars) and maize (nine cultivars) grain on nitrogen balance and apparent metabolizable energy (AME_N_) content for broiler chickens. In vivo digestibility and balance trials were carried out with 90 Ross 308 chickens (2 × 9 groups with 5 birds per group) aged from 42 to 49 days, separately for each cultivar. Considerable variation within each cereal species in fiber and non-fiber carbohydrate fractions and nutrient digestibility of grain were demonstrated. Additionally, regression equations were proposed which allow the estimation of AME_N_ content of wheat and maize grain varieties based on simple analytical procedures, including cell wall components, starch, and sugars. For practical purposes, these equations seem to be the best solution while reducing time, labor, and cost of analytical procedures.

## 1. Introduction

Recent advances in plant breeding increase differences in the chemical composition of grain between varieties within each cereal species [1,2]. In the case of wheat and maize grain, which are most often used in poultry nutrition, individual variability may differ not only in protein, starch, or fiber content but also in nutrient digestibility and metabolizable energy content [3]. Unfortunately, this fact is not widely considered during diet formulation for animals and very small varietal differences are assumed. 

A large number of new cereal varieties makes it necessary to develop simple procedures for determining their nutritional value without time-consuming and expensive studies on animals. This opportunity is provided by regression equations used for estimating digestible nutrient and metabolizable energy content based on laboratory data. The improvement of analytical procedures has contributed to the elaboration of rapid and efficient methods for the determination of nutrients, in particular the cell wall content of plant feeds [4]. The value of metabolizable energy in cereal grain is negatively correlated to crude fiber content; however, it is believed that this kind of fiber determination is not sufficiently described within the energy value for mixtures containing cereal grain [5,6]. In this context, the effect of crude, detergent, or dietary fiber content, including soluble and insoluble fractions, on energy utilization in poultry, has not been adequately studied. 

The equations for estimating the energy value of feeds for poultry are based on digestible nutrient content and fail to account for unique differences of carbohydrate structure between cereal varieties; however, some research [7,8,9] indicates a significant role of cell wall structural components in the estimation of the apparent metabolizable energy (AME_N_) value of feeds. To more accurately predict the nutritive effect of fiber from raw materials, a better characterization of fiber fractions, their degradation in the chicken, and their physiological effects are required. Therefore, we hypothesized that the inclusion of different fiber fraction content into regression equations may significantly improve the efficiency and accuracy of metabolizable energy prediction. Additionally, replacing data related to digestible nutrients with crude nutrients, especially with crude, detergent, or dietary fiber content, may facilitate the prediction procedure. The study aimed to determine the effect of nutrient content, with special emphasis on carbohydrates, on nutrient digestibility, nitrogen balance, and apparent metabolizable energy content (AME_N_) of wheat and maize grain for broiler chickens.

## 2. Materials and Methods

The study was conducted at the Department of Animal Nutrition and Biotechnology and Fisheries of the University of Agriculture in Krakow (Poland). Grain from 6 varieties of spring wheat, 3 varieties of winter wheat, and 9 varieties of maize (flint, semi flint, semi dent, and dent) were selected and prepared (dried and ground) for further procedures (Table 1). Plants were grown in the same year in the experimental farm Małopolska Plant Raising HBP Ltd. near Kraków located in south-eastern Poland. The crops were grown on Haplic Phaeozem formed from loess and classified as very good wheat complex soil. The physicochemical properties of the soil were appropriate for the habitat requirements of both species. For each wheat and maize variety, chemical analysis of grain were performed in 4 different representative samples originating from 4 different experimental crop plots.

The in vivo digestibility and balance trials were carried out separately for each variety of wheat and maize grain with 90 Ross 308 chickens, 42 to 49 days of age (2 × 9 groups with 5 birds per group in 2 replicates each; n = 80 for wheat and maize grain, separately). Before the digestibility trials, chickens were kept in pens and fed first the standard broiler starter diet (from 0 to 14 d of life) and then (from 14 to 42 d of life) they were kept in individual cages and fed diets based on the evaluated wheat or maize cultivars and containing (g/kg):

wheat 732.6, soybean meal 150, fish meal 80, monocalcium phosphate 5.8, limestone 18, sodium chloride 3.0, L-lysine 2.6, DL-methionine 3.0, and vitamin-mineral premix 5; 

maize 708.5, soybean meal 180, fish meal 80, monocalcium phosphate 5, limestone 11, L-lysine 4.7, DL-methionine 2.8, vitamin-mineral premix 5, to satisfy nutrient requirements of broilers. 

Nutritive values of mixtures within each cereal species were similar and contained an average 11.31 and 10.88 AME_N_, 20.2% and 20.8% CP, and 1.19% and 1.18% Lys, respectively, for wheat and maize. 

During the digestibility trials, chickens were kept individually in metabolic cages with free access to water. Grain was fed ad libitum as coarsely ground meal which could pass through a 4.0 mm screen sieve in 2000 rpm speed in hammer mill. Both pre-treatment and data collection periods lasted 4 days (in total, 8 days per trial). Excreta were collected twice a day from trays placed under each cage and stored at −18 °C. All methods and procedures in this study were approved and followed the recommendations of the Local Ethics Committee in Krakow (Poland).

Prior to chemical analysis, air-dried samples of wheat and maize grain were ground to pass through a 1 mm sieve with a Pulverisette 15 Laboratory Cutting Mill (Fritsh GMBH, Idar-Oberstein, Germany) and analyzed for content of dry matter (DM), ash, crude protein (CP), ether extracts (EE), and crude fiber (CF) using standard analytical procedures (procedure nos. 934.01, 942.05, 976.05, 920.39, and 962.09, for DM, ash, CP, EE, and CF, respectively; [10]. Neutral detergent fiber determined with heat-stable amylase (aNDF) [11], acid detergent fiber (ADF) [10]; official method 973.18 and acid detergent lignin (ADL) [4] were determined using an Ankom220 Fiber Analyzer (Ankom Technology, NY, USA). The starch content was determined by an enzymatic method [12]. The same procedures were used for chemical analyses of the excreta. Gross energy (GE) content was determined using a bomb calorimeter (KL-10, PRECYZJA, Bydgoszcz, Poland). The content of dietary fiber (soluble—SDF and insoluble—IDF) was determined based on 991.43 AOAC [10] procedure [13]. Water-soluble carbohydrates were analyzed spectrophotometrically using the color reaction with anthrone in concentrated, purified H_2_SO_4_. Prior to measurements, the samples were deproteinized using zinc acetate Zn(CHCOO)_2_·H_2_O 275.12 g/L^−1^ water and potassium ferrocyanide K_4_Fe(CN_6_) 3H_2_O 171.99 g/L^−1^ water. Extinction was measured at a wavelength of λ = 620 nm [14]. The proportion of amylose and amylopectin in starch was determined following the method described by Morrison et al. [15]. 

The results related to the chemical composition of feeds and excreta were used to calculate the coefficients of dry matter, organic matter, ether extracts, and N-free extract apparent digestibility. The apparent crude protein digestibility was calculated using the alpha-amino nitrogen (N-α-NH_2_) method [16] modified by Barteczko et al. [17]. This method is based on the determination of alfa-amino groups in the feces from undigested feed proteins. The first stage is the hydrolysis of feed protein and feces in hydrochloric acid (HCl). Subsequently, the feces samples are subjected to pressure distillation to remove the non-protein nitrogen fraction (mainly ammonia-NH_3_), which can positively react with ninhydrin and thus affect the results. Hydrolysates of the distillation feces and the corresponding feed have undergone a reaction during which free amino groups formed a colored complex with ninhydrin. Next, the extinction of fecal samples and corresponding feed samples were measured at the wavelength of 570 nm.

Nitrogen balance (BN), called also as “N retained”, was calculated according to the formula:

BN (g) = dietary N intake − (fecal N + urine N)(1)

The metabolizable energy value corrected to zero nitrogen balance (i.e., to the nitrogen equilibrium of the birds; AME_N_) was calculated according to the formula:

AME_N_ (kcal∙kg^−1^ converted to MJ∙kg^−1^ DM) = AME − (BN g × 8.73) (2)

where: AME (kcal∙kg^−1^) = GE − E_excreta_ − E_urine_ (3)

The regression equations for the provision of AME_N_ content were estimated using multiple forward stepwise regression [18]. The significance of model parameters was analyzed using Student’s t-test. The coefficient of determination (R^2^), the standard deviation of the difference between actual and estimated values (RSD), and mean estimation error (S) were also considered. The results of chemical analysis and digestibility trials were analyzed statistically by one-way ANOVA and Tukey’s range test [18]. The main experimental factor for wheat grains included the differences between winter and spring varieties while for maize grains included the differences between early, medium early, and medium late forms of varieties. The significance level was set at *p* ≤ 0.05. Tendencies were discussed at 0.05 < *p* < 0.10 unless otherwise stated. All data are reported as least squares means with a pooled standard error of the means.

## 3. Results

### 3.1. Chemical Composition of Wheat and Maize Variety

Large differences between wheat varieties were found for nutrients, gross energy, and AME_N_ content in grains (Table 2). Crude protein content ranged from 123 to 155 g/kg DM with significant differences between spring and winter form (*p* < 0.05). Variation range for ether extracts and starch content was ±8.0 g/kg DM and ±46.9 g/kg DM, respectively (*p* < 0.05). Wheat varieties differed significantly in the content of IDF (±8.3 g/kg DM) and SDF (±7.6 g/kg DM). The differences in AME_N_ content (*p* < 0.05) fluctuated within ±1.83 MJ.

Grain from maize varieties (Table 3) differed considerably in CP (±18.0 g/kg DM) and ether extracts content (±10.9 g/kg DM), with significant differences between early, medium early, and medium late form (*p* < 0.05). Differences in starch content among varieties amounted to 10 percentage units. Varieties richest in starch had the lowest CP content. Considerable variation was shown in the content of SDF (±6.2 g/kg DM) and AME_N_ (±0.5; *p* < 0.05).

### 3.2. Nutrient Digestibility

Variety had an effect (*p* < 0.05) on the coefficient of nutrient digestibility of wheat grain (Table 4). The average coefficient of CP digestibility was 76.41% (±4.98) (*p* < 0.05). The N-free extract (NFE) digestion exceeded 90.0%, except the Bryza and Vinjett varieties. 

The highest CP digestibility of maize grain was found for Boruta and Arobase, and the lowest for the Moncada variety (*p* < 0.05; Table 5). The average digestibility coefficient of ether extracts was 67.4%, including variation of ±15% within varieties (*p* < 0.05). NFE digestion averaged 93%, with inter-form differences of five percentage units (*p* < 0.05). 

### 3.3. Estimation of Apparent Metabolizable Energy Content

In order to choose the most predictive models, we used the highest predicted R-squared and the lowest RSD. The regression equation for estimating AME_N_ content of wheat grain, which contained only basic crude nutrients, was not significant (*p* = 0.18) and explained only 23% of the dependent variable variation (data not presented). The inclusion of the variables related to detergent fiber (ADL, ADF, and aNDF), starch and sugars (Table 6) to the model increased estimation accuracy significantly (R^2^ = 0.92; *p* < 0.01). Further inclusion of CP, NFE, and IDF into the list of independent variables slightly improved estimation by reducing RSD (0.24 vs. 0.20; *p* < 0.05). The latter regression equation explained 95% of the dependent variable variation, and the discrepancy between empirical values and those predicted by the model were 0.19 MJ. The estimate of AME_N_ content of maize grain (Table 6) based on basic nutrient content alone was relatively low. Combining into this model the content of different carbohydrates significantly increased estimation accuracy. Similarly, the use of soluble and insoluble dietary fiber content in the model alone did not give satisfactory results (R^2^ = 0.85; RSD = 0.20). Finally, the inclusion of detergent fiber, IDF, SDF, starch, and sugars in the list of independent variables increased estimation accuracy significantly (R^2^ = 0.98; RSD = 0.10), which explained 97% of the dependent variable variation and had the smallest estimation error.

## 4. Discussion

### 4.1. Nutritive Value of Wheat and Maize Grain

Maize and wheat grain are the main sources of energy for broiler chickens due to the high content of readily available carbohydrates. The nutritive value of these grains depends not only on nutrient content and digestibility but also on the concentration of anti-nutritional substances, of which, the most important is soluble dietary fiber [5]. Solubilization is a prerequisite for fermentation, but even if solubilized during the digestive processes, a substantial part of non-starch polysaccharides (NSP) may remain undegraded. Other possible limiting factors for NSP degradation could be physical entanglement of polysaccharides in the cell wall matrix, time available for fermentation and finally the absence of appropriate enzyme activities as determined by the microbial colonization in the gastrointestinal tract [19,20]. 

In the present study, the evaluation of the nutritive value of wheat varieties showed substantial differences in the chemical composition of grain. Wheat varieties evaluated by Gutiérrez-Alamo et al. [21] contained from 7.5% to 13.1% CP. Such large differences in CP content between different batches of wheat grain were affected by genotype and environmental conditions. In our study, these last factors were largely eliminated by using grain grown in the same cultivation area and harvested during the same year. 

Studies carried out thus far on the nutritive value of wheat grain on nutrient utilization by broiler chickens focus mostly on the effects of total dietary fiber (TDF), non-starch polysaccharides (NSP), and non-cellulose polysaccharides [21,22]. TDF content of wheat grain depends on its genotype, cultivation area, and climatic conditions [2,23]. Perhaps for this reason the variation in soluble and insoluble dietary fiber content determined in our study were higher than those reported by Steenfeldt et al. [22]. 

It is worth emphasizing that other analytical procedures for determination of cell wall components in animal feedstuffs become more and more available. For example, detergent fiber fraction content is routinely determined in ruminant feeds but sporadically in feeds for monogastric animals. The classification of total content of cell wall components (aNDF) into partially digested acid detergent fraction (ADF) and indigestible acid detergent lignin (ADL) is connected with the unique characteristics of ruminant digestion [4]. Jamroz et al. [19] stated that in 42-day-old broiler chickens, the digestibility coefficients were about 35% for NDF and 2% for ADF. The fact that the analysis of detergent fiber content is easy and relatively inexpensive to perform compared to dietary fiber may justify its use also in poultry or pig nutrition [21]. 

In the studies by Carré et al. [24], and Rodehutscord et al. [2], there were considerable differences in the starch content between wheat varieties. According to Gutiérrez-Alamo et al. [21] starch content, its structure, and digestibility is highly correlated to AME_N_ value and mostly related to variety. In turn, cultivars rich in NSP can increase the activity of microorganisms in the digestive tract of broiler chickens as a result of changes in the proportion of digested and undigested feed particles. Furthermore, an increase in the numbers of small intestine microflora may indirectly reduce ether extracts digestibility [6]. Maisonnier et al. [20] showed, that the increase of digesta viscosity, as a result of feeding large amounts of wheat grain in the diet (over 50%) and unfavorable fatty acid profile of wheat fat, are the main reasons for the low digestibility of ether extracts. In our studies, the low ether extracts content in wheat grain varieties was associated with its low digestibility.

The age of birds may be particularly important in the determination of the energy value of cereal grains. Along with the development of the gastrointestinal tract and its ability to digest nutrients, the use of feed energy increases. The main reason for the variability in the use of feed energy is the content of fat in them, which young birds digest worse than adult birds. This results mainly from the insufficient bile secretion and intestinal flora composition. Simultaneously with age, the sensitivity of the organism to the anti-nutritive factors contained in the feed including fiber changes [19]. Differences in the AME_N_ content in the grain between different varieties of cereals of the same species are generally lower when tested on adult birds [25], but can be significant when carried out on broiler chickens [26]. If this variability is not taken into account, it may reduce production in young birds. A negative correlation between the energy value of cereals and the content of insoluble NSPs and their viscosity was determined in chickens [27]. In part, this may be due to the difficult access of digestive enzymes to protein and starch associated with NSP, as well as the negative effect of intestinal viscosity on the digestion of nutrients and the use of energy by young birds [5].

The AME_N_ value of wheat grain varies according to variety and the content of CP, ether extracts and starch [22,24]. In our study, the highest AME_N_ value was found in wheat varieties with the highest ether extracts and CP content. According to Steenfeldt [22], the AME_N_ value of wheat grain is affected by anti-nutritive compounds such as dietary fiber and non-starch polysaccharides. In our experiment, a significant and negative correlation between ADL and AME_N_ content was observed; however there was no effect of soluble digestible fiber (SDF) on AME_N_ value. AME_N_ content in wheat also depends on the physical characteristics of the grain, such as viscosity of the water extract, grain hardness [24], or thousand grain weight [2]. Another factor affecting AME_N_ may be grinding intensity. According to Smulikowska [5], the AME_N_ value of wheat grain was 13.7 for broilers and 15.1 MJ AME_N_/kg DM for adult cockerels, which indicates that dietary energy content depends also on the age of birds. 

Maize grain is relatively low in CP, high in ether extracts, and characterized by higher AME_N_ content for broiler chickens as compared to other cereals [28]. In maize varieties evaluated by Rodehutscord et al. [2], the average content of ether extracts was slightly higher than found in the present study. Song et al. [3] observed considerable differences in ether extracts content between conventional maize varieties and maize with a higher content of ether extracts (over 60.0 g/kg DM). The starch content of maize grain and the proportion of amylose and amylopectins in starch depends on the variety [2] which was confirmed by our results. The amylose to amylopectin ratio influences the level of maize starch digestion. The higher the proportion of amylose in starch, the higher the digestibility and utilization of starch [29]. 

Compared to other cereal species, maize grain contains less crude fiber, non-starch, and non-cellulose polysaccharides and thus these substances have less negative effect on digestion [29]. Therefore in chickens, maize nutrients are generally characterized by high digestibility [9]. In our study, the lowest coefficients of digestibility were found for ether extracts, especially for the varieties low in ether extracts, which could result in a low daily intake of fat. 

The energy value of maize grain is higher compared to other cereals. According to Weurding et al. [30], starch provides about 60% of metabolizable energy (AME_N_) in poultry feeds. The AME_N_ content in maize grain found in the present study was in line with these findings and similar to the results reported by Sauvant, Perez, and Tran [28]. In turn, Lessire et al. [7] found a positive correlation between GE or AME_N_ and ether extracts content in maize grain.

### 4.2. Prediction of Energy Value

In most used recommendations, the energy value of feeds for poultry is calculated on the basis of basic digestible nutrient content [31]. In the present study, digestible nutrients were not included in the regression equations because it is easier and more practical to manipulate with crude nutrient content. Considering this approach, most proposed equations [31,32] are based on the content of CP, ether extracts and NFE. Crude fiber content was included only in the model for oat grain evaluation, and the effect of this nutrient has not been taken into account in the equations proposed for maize or wheat [31]. Without a doubt, crude fiber is an important component that affects the utilization of energy from a feed of plant origin but due to its low sensitivity should be replaced by other methods of fiber analysis. 

Gutiérrez-Alamo et al. [21] provided evidence that metabolizable energy value of poultry feedstuffs is more correlated to the content of some structural fractions of cell walls than to the crude fiber content of the diet. This observation is supported by our findings. The inclusion of detergent or dietary fiber content into regression equations, as independent variables, increased the accuracy of AME_N_ estimation. The prediction equation proposed by Mariano et al. [33] using differentiated meta-analysis, which accounted for EE, ash, CF, and NDF, was more efficient. According to Alvarenga et al. [34], the equation to predict AME_N_ according to content of EE, ash, CF, and NDF was the most applicable for the prediction of the energy values of feedstuffs and diets used in the poultry feed industry. Zhao et al. [8] developed regression equations which, with adequate accuracy, estimated AME_N_ of maize grain for ducks based on NDF and GE content. Also, De Oliveira and Warpechowski [9] demonstrated the significance of NDF and ADF inclusion into the regression equations for estimating AME_N_ of maize grain. Similarly, Lessire et al. [7] determined a significant effect of water insoluble cell wall component concentration in maize grain on energy utilization by cockerels. According to Meloche et al. [35], stepwise selection in multiple linear regression determined that GE, TDF, CP, and starch were the best predictors of AME_N_ in DDGS. Omission of TDF from the variable selection pool to develop a more practical model resulted in the inclusion of NDF in lieu of TDF.

Our results showed a significant effect of insoluble dietary fiber on the metabolizable energy value of maize grain; however, from a practical point of view, it seems more important to introduce content of some detergent fiber fractions (NDF, ADF, or ADL) into regression equations for estimating the energy value of feeds for poultry rather than dietary fiber fractions due to much lower cost of analysis. In this regard, the content of other carbohydrates may also be useful. The equations proposed by Lessire et al. [7] for AME_N_ prediction of wheat grain included the concentration of starch and water-soluble carbohydrates as independent variables, which significantly improved the accuracy of AME_N_ estimation. Likewise, equations developed by NRC [32] and Carré et al. [24] also used the aforementioned nutrients, which is logical since starch is the main energy component of cereal grain. 

In our study we managed to achieve R^2^ = 0.95 with RSD = 0.20 when estimating AME_N_ for wheat (Equation (2)) and R^2^ = 0.98 with RSD = 0.10 for maize (Equation (2)). As reviewed in the study of Alvarenga et al. [34], in which various equations for estimation of the AME_N_ of feedstuffs [33,36,37] were compared, the equation AME_N_ (DM basis) = 4164.187 + 51.006 ether extract − 197.663 crude ash − 35.689 crude fiber − 20.593 aNDF (R^2^ = 0.75), proposed by Mariano et al. [33] was the most applicable for the prediction of the energy values of feedstuffs and diets used in the poultry feed industry. 

The use of proven, reliable, and low-cost analytical methods will allow for the ongoing evaluation of the nutritional value of feed for poultry, especially for cereal grain. In this context, our own research indicated the need to develop these equations which will allow the estimation of the nutritive value of different cereal grain varieties based on simple analytical procedures, including cell wall components. Fiber in poultry nutrition is usually associated with reduced energy availability due to its minor role in energy supply and interference with digestive processes. A better understanding on the relation between specific fiber fractions and factors as nitrogen balance or metabolizable energy content could help to develop nutritional strategies to enhance performance and health of broiler chickens. 

Certainly, additional research is required to characterize the effect of hydrothermal processing (HP) widely used in the feed industry, including pelleting, extrusion or expansion on nutrient availability and energy value of wheat and maize grain for broiler chickens. According to recent findings [38] the effect of such treatments is equivocal and their impact on starch, fat, or protein utilization can be negligible. The beneficial impact of HP on nitrogen utilization is probably through protein denaturation and destruction of anti-nutritional factors. On the other hand, the harmful effect of HP processing on thermolabile nutrients cannot be ignored as well as on an increase of fiber solubility and digesta viscosity which could lead to a negative impact on nutrient digestibility. 

## 5. Conclusions

The chemical composition of wheat and maize grain showed large differences between varieties within each grain species. In some cases, the differences found within varieties were greater than between the cereal species commonly used in the nutrition of broiler chickens. A direct relationship between crude nutrient content, nutrient digestibility, and apparent metabolizable energy value (AME_N_) of wheat and maize grain was shown and, on this basis, regression equations for provision of AME_N_ were proposed including detergent fiber fractions, starch, and sugar concentrations. For practical purposes, these equations seems to be the best solution while reducing time, labor, and cost of analytical procedures. 

## Figures and Tables

**Table 1:** Description of wheat and maize cultivars.

Species	Form	Cultivar	Characteristics ^1^
Wheat	Spring	Bombona	Elite
		Bryza	Qualitative
		Napola	Qualitative
		Torka	Elite
		Vinjett	Elite
		Zebra	Elite
	Winter	Mikula	Fodder
		Muza	Qualitative
		Satyna	Fodder
Maize	Early (FAO 230)	Boruta	semi dent
		Pioneer PR39G12	Flint
	Medium early (FAO 230–240)	Eurostar	semi dent
		Nysa	semi dent
		Opoka	semi dent
		Pioneer PR39H84	Dent
		Smok	semi flint
	Medium late (FAO 250–260)	Arobase	semi flint
		Moncada	semi flint

^1^ technological group (wheat) or type of endosperm (maize).

**Table 2:** Chemical composition (g/kg, dry matter—DM), gross energy (GE), and apparent metabolizable energy content (AME_N_) of wheat grain.

Item	Spring Cultivars							Winter Cultivars						SEM	*p*-Value (Spring vs. Winter)
	Bombona	Bryza	Napola	Torka	Vinjett	Zebra	Mean	SD	Mikula	Muza	Satyna	Mean	SD		
Dry Matter, g/kg	867	868	866	863	873	863	867	3.6	862	871	856	863	6.9	2.8	0.11
TGW ^1^	42	42	44.1	42	40	41	42	1.4	52	44	43	46	4.3	1.8	<0.01
Crude ash	19	20	20	20	18	19	19	0.7	17	18	19	18	0.6	0.2	<0.01
Crude protein	137	139	147	123	155	138	140	10.1	126	136	125	129	5.2	2.1	0.03
Ether extracts	20	14	22	20	20	21	19	2.7	14	15	16	15	1.0	0.4	<0.01
NFE ^2^	797	805	789	815	778	800	797	12.3	818	804	813	812	6.3	2.6	0.02
Sugars	71	47	52	48	61	47	54	9.3	34	38	71	48	18.2	7.4	0.32
Starch	614	601	673	748	650	662	658	49.6	666	724	719	703	28.9	12.0	0.06
Amylose	258	243	263	268	255	253	257	8.0	266	255	249	257	7.6	3.1	0.95
Amylopectin	356	357	410	480	395	409	401	43.4	400	469	470	446	36.0	14.7	0.04
Amylose in starch %	42	41	39	36	39	38	39	2.0	40	35	35	37	2.6	1.1	0.03
Crude fiber	27	23	22	22	30	23	24	3.1	24	27	28	26	1.6	0.7	0.19
aNDF ^3^	130	112	126	105	113	116	117	8.8	114	115	132	120	9.2	3.7	0.52
ADF ^4^	40	34	43	37	42	41	39	3.3	37	40	43	40	2.7	1.1	0.74
ADL ^5^	0.6	0.1	0.1	0.1	1.4	0.4	0.5	0.6	0.2	0.8	0.6	0.5	0.3	0.1	0.40
IDF ^6^	107	106	117	108	107	116	110	4.6	115	106	131	118	11.4	4.6	0.07
SDF ^7^	19	19	37	22	18	18	22	7.2	24	14	10	16	6.4	2.6	<0.01
TDF ^8^	126	125	154	130	125	134	132	11.2	139	120	141	133	11.6	3.8	0.19
GE, MJ/kg DM	18.5	18.4	18.6	18.4	18.6	18.5	18.5	0.1	18.3	18.4	18.4	18.4	0.0	0.0	<0.01
AME_N_, MJ/kg DM	14.6	14.6	15.5	14.7	14.2	13.5	14.5	0.7	15.3	15.3	15.8	15.5	0.3	0.1	<0.01
AME_N_/GE %	78.9	76.7	83.2	80.0	76.3	73.0	78.3	3.7	83.2	83.4	85.9	84.1	1.4	0.6	<0.01

^1^ thousand grain weight, ^2^ N-free extract, ^3^ neutral detergent fiber determined with heat-stable amylase, ^4^ acid detergent fiber, ^5^ acid detergent lignin, ^6^ insoluble dietary fiber, ^7^ soluble dietary fiber, ^8^ total dietary fiber.

**Table 3:** Chemical composition (g/kg DM), gross energy (GE), and apparent metabolizable energy content (AME_N_) of maize grain.

Item	Early (FAO <230)				Medium Early ( FAO 230–240)							Medium Late (FAO 250–260)				SEM	*p*-Value (FAO)
	Boruta	Pioneer G12	Mean	SD	Eurostar	Nysa	Opoka	Pioneer H84	Smok	Mean	SD	Arobase	Moncada	Mean	SD		
Dry matter g/kg	878	871	875	3.6	870	882	871	877	888	878	7.0	881	878	880	1.8	0.9	0.45
Crude ash	12	12	12	0.1	13	14	13	11	16	14	1.7	14	15	15	0.3	0.2	0.07
Crude protein	110	99	104	6.2	93	130	100	91	137	110	20.3	127	90	109	21.1	10.6	0.88
Ether extracts	47	47	47	0.1	50	45	47	19	38	40	11.7	50	27	39	13.2	6.6	0.53
NFE ^1^	811	818	815	3.8	822	792	823	857	788	816	26.5	783	843	813	35.2	17.6	0.97
Sugars	31	15	23	9.4	16	18	12	13	16	15	2.4	15	8	12	4.1	2.0	0.02
Starch	678	766	722	51.0	744	675	725	757	680	716	34.7	665	753	709	50.9	25.5	0.91
Amylose	229	215	222	8.4	212	215	226	223	220	219	5.4	215	219	217	2.9	1.4	0.51
Amylopectin	449	552	500	59.4	532	460	499	533	460	496	34.2	450	534	492	48.1	24.0	0.96
Amylose in starch %	34	28	31	3.3	29	32	31	30	32	31	1.5	32	29	31	1.8	0.9	0.98
Crude fiber	20	24	22	2.5	22	19	17	22	22	20	2.4	26	24	25	1.1	0.6	0.01
aNDF ^2^	136	117	127	10.9	155	117	104	137	156	134	21.8	159	140	149	11.1	5.6	0.21
ADF ^3^	48	54	51	3.4	47	45	40	46	46	45	2.7	65	49	57	9.5	4.6	<0.01
ADL ^4^	9	15	12	3.4	6	9	8	8	6	7	1.2	12	9	10	2.0	1.0	<0.01
IDF ^5^	93	85	89	4.5	107	100	97	103	94	100	4.7	107	119	113	7.0	3.5	<0.01
SDF ^6^	7	9	8	1.1	9	8	4	8	6	7	2.0	9	8	8	0.8	0.4	0.31
TDF ^7^	100	94	97	4.2	116	108	101	111	100	107	6.8	116	127	122	7.7	3.8	0.07
GE, MJ/kg DM	18.8	19.0	18.9	0.2	19.1	18.7	18.6	18.6	18.8	18.8	0.2	18.9	18.3	18.57	0.4	0.2	0.14
AME_N_, MJ/kg DM	15.9	16.3	16.1	0.2	15.7	15.3	15.4	15.6	15.5	15.5	0.2	15.3	14.5	14.9	0.5	0.2	<0.01
AME_N_/GE %	84.8	85.6	85.2	0.5	82.2	81.8	82.7	84.0	82.5	82.7	0.9	81.0	79.2	80.09	1.0	0.5	<0.01

^1^ N-free extract, ^2^ neutral detergent fiber determined with heat-stable amylase, ^3^ acid detergent fiber, ^4^ acid detergent lignin, ^5^ insoluble dietary fiber, ^6^ soluble dietary fiber, ^7^ total dietary fiber.

**Table 4:** Apparent nutrient digestibility (%), nitrogen balance (NB g), and nitrogen retention (NR %) of wheat grain in broilers.

Item	Spring Cultivars								Winter Cultivars					SEM	*p*-Value (Spring vs. Winter)
	Bombona	Bryza	Napola	Torka	Vinjett	Zebra	Mean	SD	Mikula	Muza	Satyna	Mean	SD		
Digestibility coefficient															
Dry matter	79.6	76.1	83.9	80.2	74.3	71.6	77.6	4.3	82.7	82.8	86.2	83.9	1.92	0.8	<0.01
Organic matter	83.7	79.8	87.1	83.3	79.3	75.1	81.4	4.1	85.6	86.0	88.8	86.8	1.6	0.6	0.01
Ether extracts	69.7	49.9	82.3	48.2	53.0	48.9	58.7	14.1	61.0	60.8	85.7	69.2	13.1	5.4	0.14
NFE ^1^	92.5	88.9	94.2	92.7	89.6	82.1	90.0	4.2	93.1	94.1	94.3	93.8	0.6	0.2	0.05
Crude protein	77.3	76.6	81.6	72.5	73.0	70.3	75.2	4.6	76.2	81.5	83.9	80.6	3.7	1.5	0.02
NB g	1.6	1.4	2.2	1.2	1.5	1.8	1.6	0.3	1.7	1.8	2.0	1.8	0.2	0.1	0.25
NR %	49.1	43.0	59.7	42.5	45.4	49.2	48.1	7.2	52.2	50.8	64.5	55.9	7.3	3.0	0.05

^1^ N-free extract.

**Table 5:** Apparent nutrient digestibility (%), nitrogen balance (NB g), and nitrogen retention (NR %) of maize grain in broilers.

Item	Early (FAO 230)				Medium Early (FAO 230–240)								Medium Late (FAO 250–260)				SEM	*p*-Value (FAO)
	Boruta	Pioneer G12	Mean	SD	Eurostar	Nysa	Opoka	Pioneer H84	Smok		Mean	SD	Arobase	Moncada	Mean	SD		
Digestibility coefficient																		
Dry matter	85.6	85.6	85.9	0.3	81.7	82.4	82.9	84.1		83.3	82.9	1.2	81.3	80.7	81.0	0.8	0.4	<0.01
Organic matter	87.5	87.6	87.6	0.3	84.2	84.7	85.1	86.2		85.6	85.2	1.1	83.8	83.3	83.5	0.7	0.3	<0.01
Ether extracts	73.8	79.6	76.7	3.5	77.8	67.1	76.9	41.0		69.0	66.4	14.3	77.2	37.7	57.5	23.0	7.5	0.23
NFE^1^	94.5	93.6	94.0	0.7	91.7	95.0	91.6	92.8		95.5	93.3	1.8	92.1	91.2	91.6	0.	0.3	0.08
Crude protein	82.0	81.7	81.9	1.1	77.9	75.0	77.3	81.5		76.2	77.6	3.1	72.5	79.6	76.0	4.7	2.3	0.05
NB g	1.1	1.0	1.1	0.1	0.8	0.9	1.1	0.9		1.2	1.0	0.2	1.2	0.7	0.9	0.3	0.1	0.75
NR %	51.3	52.5	51.9	1.6	40.0	39.9	51.3	49.3		52.1	46.5	8.6	48.7	38.8	43.7	6.8	3.4	0.30

^1^ N-free extract.

**Table 6:** Chosen regression equations for prediction of AME_N_ (kcal∙kg^−1^) of wheat and maize grain from crude nutrient content *.

Item (Equation no **)	Constants	CP ^2^	ADL ^3^	ADF ^4^	aNDF ^5^	NFE ^6^	IDF ^7^	SDF ^8^	Starch	Sugars	R^2 (9)^	RSD ^10^	*p*-Value
Wheat (1)	3.75		−0.35	0.04	0.06				0.006	−0.02	0.92	0.24	<0.01
B ^1^	1.3		0.05	0.03	0.01				0.001	0.01			
*p*-Value	0.01		<0.01	0.28	<0.01				<0.01	0.01			
Wheat (2)	−111.54	0.1	−0.39	0.28	0.02	0.13	−0.03				0.95	0.20	<0.01
B ^1^	25.41	0.03	0.04	0.04	0.01	0.03	0.01						
*p*-Value	<0.01	<0.01	<0.01	<0.01	0.02	<0.01	0.02						
Maize (1)	19.24						−0.05	0.11			0.85	0.20	<0.01
B ^1^	0.54						0.001	0.03					
*p*-Value	<0.01						<0.01	<0.01					
Maize (2)	13.49		−0.39	0.16	−0.04		−0.06	0.14	0.01	0.03	0.98	0.10	<0.01
B ^1^	1.05		0.07	0.03	0.01		0.004	0.03	0.01	0.03			
*p*-Value	<0.01		<0.01	<0.01	<0.01		<0.01	<0.01	<0.01	<0.01			

* Content of nutrients expressed in g/kg DM, ^1^ standard error of estimate, ^2^ crude protein, ^3^ acid detergent lignin, ^4^ acid detergent fiber, ^5^ neutral detergent fiber determined with heat-stable amylase, ^6^ N-free extract, ^7^ insoluble dietary fiber, ^8^ soluble dietary fiber, ^9^ coefficient of determination, ^10^ residual standard deviation. ** An example of equation structure for wheat grain (1) is: AME_N_ = 3.75 − 0.35ADL + 0.04ADF + 0.06aNDF + 0.006Starch − 0.02Sugars.
